# Next-Generation Sequencing of the Whole Bacterial Genome for Tracking Molecular Insight into the Broad-Spectrum Antimicrobial Resistance of *Helicobacter pylori* Clinical Isolates from the Democratic Republic of Congo

**DOI:** 10.3390/microorganisms8060887

**Published:** 2020-06-11

**Authors:** Evariste Tshibangu-Kabamba, Patrick de Jesus Ngoma-Kisoko, Vo Phuoc Tuan, Takashi Matsumoto, Junko Akada, Yasutoshi Kido, Antoine Tshimpi-Wola, Pascal Tshiamala-Kashala, Steve Ahuka-Mundeke, Dieudonné Mumba Ngoy, Ghislain Disashi-Tumba, Yoshio Yamaoka

**Affiliations:** 1 Department of Environmental and Preventive Medicine, Faculty of Medicine, Oita University, Oita 879-5593, Japan; evaristetshibangu@gmail.com (E.T.-K.); vophuoctuandr@gmail.com (V.P.T.); tmatsumoto9@oita-u.ac.jp (T.M.); akadajk@oita-u.ac.jp (J.A.); yasutoshikido@gmail.com (Y.K.); 2 Department of Internal Medicine, Faculty of Medicine, University of Mbujimayi, Mbujimayi, DR Congo; tdisashi@yahoo.fr; 3 Department of Internal Medicine, Gastroenterology and Hepatology Section, Faculty of Medicine, University of Kinshasa, Kinshasa, DR Congo; patrickdejesus3@gmail.com (P.d.J.N.-K.); antshimpi@aol.com (A.T.-W.); 4 Department of Gastroenterology and Hepatology, Cinquantenaire’s Hospital, Kinshasa, DR Congo; 5 Department of Internal Medicine, Gastroenterology and Hepatology Section, General Referential Hospital of Bukavu, DR Congo; 6 Department of Endoscopy, Cho Ray Hospital, Ho Chi Minh 70000, Vietnam; 7 Department of Parasitology, Osaka City University, Osaka 545-8585, Japan; 8 Department of Internal Medicine, Gastroenterology and Hepatology Section, Marie-Yvettes Clinics, Kinshasa, DR Congo; 9 Department of Internal Medicine, Gastroenterology and Hepatology Section, Astryd Clinics, Kinshasa, DR Congo; ptshiamala@yahoo.fr; 10 Department of Virology, National Institute of Biomedical Research, Kinshasa, DR Congo; amstev04@yahoo.fr; 11 Department of Parasitology, National Institute of Biomedical Research, Kinshasa, DR Congo; mumbadieudonne@yahoo.fr; 12 Department of Tropical Medicine, School of Medicine, University of Kinshasa, Kinshasa, DR Congo; 13 Department of Medicine, Gastroenterology and Hepatology Section, Baylor College of Medicine, Houston, TX 77030, USA

**Keywords:** *Helicobacter pylori*, whole genome sequencing, drug resistance, antimicrobial susceptibility testing, next-generation sequencing, Democratic Republic of Congo, Africa

## Abstract

Antimicrobial susceptibility testing (AST) is increasingly needed to guide the *Helicobacter pylori* (*H. pylori*) treatment but remains laborious and unavailable in most African countries. To assess the clinical relevance of bacterial whole genome sequencing (WGS)-based methods for predicting drug susceptibility in African *H. pylori*, 102 strains isolated from the Democratic Republic of Congo were subjected to the phenotypic AST and next-generation sequencing (NGS). WGS was used to screen for the occurrence of genotypes encoding antimicrobial resistance (AMR). We noted the broad-spectrum AMR of *H. pylori* (rates from 23.5 to 90.0%). A WGS-based method validated for variant discovery in AMR-related genes (discovery rates of 100%) helped in identifying mutations of key genes statistically related to the phenotypic AMR. These included mutations often reported in Western and Asian populations and, interestingly, several putative AMR-related new genotypes in the *pbp1A* (e.g., T558S, F366L), *gyrA* (e.g., A92T, A129T), *gyrB* (e.g., R579C), and *rdxA* (e.g., R131_K166del) genes. WGS showed high performance for predicting AST phenotypes, especially for amoxicillin, clarithromycin, and levofloxacin (Youden’s index and Cohen’s Kappa > 0.80). Therefore, WGS is an accurate alternative to the phenotypic AST that provides substantial decision-making information for public health policy makers and clinicians in Africa, while providing insight into AMR mechanisms for researchers.

## 1. Introduction

Gastrointestinal diseases and gastric cancer constitute an important public health problem worldwide [1]. In the last decades, the discovery of the role of *Helicobacter pylori* (*H. pylori*) and Epstein–Barr virus (EBV) infections in the development of gastric diseases have raised these pathogens as the main modifiable factors for preventing gastric cancer [2,3,4]. Particularly, the *H. pylori* eradication treatment can heal or prevent related complications, including gastritis, peptic ulcer disease, and distal gastric cancer [3,4,5]. However, only a few antimicrobials are effective against *H. pylori*, such as amoxicillin (AMX), clarithromycin (CLA), metronidazole (MTZ), and levofloxacin (LEVO), and combination therapies that consist of two or three antibiotics, an acid inhibitor, and/or a bismuth component are required [4,6]. In recent years, the extensive use and limited choice of effective antimicrobials coupled with the exceptional adaptation abilities of *H. pylori* have resulted in the development of antimicrobial resistance of the species, posing a serious threat to currently available treatment options [7]. Standard antimicrobial susceptibility testing (AST) has become increasingly needed for guiding decisions about appropriate therapies. The standard AST of *H. pylori* is a culture-based method that is typically fastidious (slow bacterial growth), time-consuming (delivering results in around two weeks), expensive, and technically challenging; moreover, it provides outcomes that are susceptible to an inter-observer variability [8,9]. These factors emphasize the need for more rapid and cost-effective molecular methods that can enable a reliable prediction of the phenotypic antimicrobial resistance (AMR).

Recently, bacterial whole genome sequencing (WGS) enabled by next-generation sequencing (NGS) technologies has emerged as a cost-effective, powerful, and fast tool for AMR prediction and infectious disease surveillance [10,11,12,13]. Within a clinically relevant timeframe (24 to 72 h), WGS can provide a comprehensive view of bacterial genotypes including eventual AMR-related genetic determinants [12,14,15]. WGS-based methods are particularly relevant for tracking complex or nested genetic factors driving AMR and offer interesting potential for the discovery of novel or rare genotypes encoding AMR in clinical isolates [10,12,16]. However, beyond the challenge of analyzing high-throughput data arising from NGS, there is still a need for the standardization of WGS approaches and pipelines that are in use for detecting AMR-related determinants [10,12,14,16]. Nevertheless, the application of WGS remains an attractive option for genotyping the AMR of *H. pylori* species in diagnostic microbiology laboratories, especially in Africa. This continent faces important challenges in controlling *H. pylori*, given the high prevalence of infections and high rates of AMR [17,18]. While there are no local *H. pylori* treatment guidelines in most African countries, the implementation of a standard AST before drug prescription remains difficult [17]. Furthermore, since Africa is colonized by specific *H. pylori* genetic populations [3,19], strains circulating in different regions may have their own genetic background preference to develop AMR, which would differ from the strategies displayed by the non-African species. Therefore, the molecular methods to be used for tracking *H. pylori* AMR in Africa should be able to detect new resistance genotypes while identifying local high-confidence genetic determinants to efficiently predict the phenotypic AST.

In the present study, we aimed to assess the phenotypic AMR, identify related genetic determinants, and explore the feasibility of genomic NGS-based approaches for tracking resistance in *H. pylori* clinical isolates from the Democratic Republic of Congo (DRC) in Middle Africa. Therefore, we have reported the first local data related to *H. pylori* AMR showing the broad-spectrum resistance of isolates that likely supports AST before treatment in a clinical practice. We have also successfully screened massive WGS data using a validated approach to detect AMR-related genetic features and uncover putative new mechanisms of clinical resistance in *H. pylori*. Finally, we discussed the usefulness of genomics for tracking AMR in comparison to classical PCR-based molecular methods and phenotypic AST.

## 2. Materials and Methods

### 2.1. Patients and Biological Samples

One hundred and nine *H. pylori* clinical strains were isolated from 220 patients undergoing upper gastro-duodenal endoscopy between August 2017 and October 2018 in four hospitals (the Cinquantenaire’s Hospital, Astryd Clinics, Marie-Yvette Clinics, and The Presidential Hospital of the African United City) located in Kinshasa, the capital city of the DRC. Gastric biopsy specimens sampled from the antrum were cultured following a previously established standard procedure [20]. Briefly, biopsy specimens were transitorily stored at −80 °C at the National Institute of Biomedical Research (INRB) in Kinshasa. Then, they were shipped to Oita University in Japan, where all analyses were performed. Upon arrival, each specimen was inoculated onto a *Helicobacter* selective Agar medium (Nissui Pharmaceutical co., Ltd., Tokyo, Japan) and incubated for up to 10 days. *H. pylori* colonies growing on the selective plates were identified and sub-cultured for three to four days on Brucella Agar plates (Becton Dickinson, Sparks, MD, USA) supplemented with 7% horse blood (Nippon Biotest Laboratories Inc., Tokyo, Japan). Bacterial cultures were maintained at 37 °C in microaerophilic conditions (10% CO_2_, 5% O_2_, and 85% N_2_).

### 2.2. Phenotypic Antimicrobial Susceptibility Testing

The antimicrobial susceptibility was phenotypically assessed by the agar dilution assay using *H. pylori* colonies isolated from gastric biopsy specimens. The minimum inhibitory concentrations (MICs) of the antimicrobials were determined following the protocols of the Clinical and Laboratory Standards Institute (Wayne, PA, USA) [21]. Briefly, two-fold serial dilutions of AMX, CLA, LEVO, and MTZ (Wako Pure Chemical Industry, Osaka, Japan) were prepared in a Müller-Hinton agar supplemented with 5% horse blood. Each bacterial sample sub-cultured for three days was suspended in physiological saline, adjusted to an OD_600_ of 0.1, and spotted as a 1µL inoculum onto culture plates. The MICs of antimicrobials were determined after 72 h of incubation. The *H. pylori* strain 26695 was used as a control strain. Agar dilution assays were duplicated and repeated three times. Additionally, the antimicrobial susceptibility of *H. pylori* strains that showed discrepant results between the agar dilution assay and genetic information was assessed using the E-Test^®^ (bioMérieux SA, Lyon, France) method in culture growth using an OD_600_ of 0.2. Clinical breakpoints between resistant and susceptible strains were determined following the guidelines of the European Committee on Antimicrobial Susceptibility Testing (EUCAST) available at http://www.eucast.org/ (accessed on 6 August 2019). Finally, the production of β-lactamase by *H. pylori* strains was also tested by using the Nitrocefin BBL^TM^ DrySlide^TM^ (Becton Dickson and Company, Sparks, MD, USA) according to the manufacturer’s instructions.

### 2.3. Preparation of Genomic DNA, Library Preparation, and Whole Genome Sequencing

The total DNA of each strain was extracted using the DNeasy Blood and Tissue Kit according to the manufacturer’s guidelines (Qiagen, Hilden, Germany). The DNA concentration of each sample was quantified using the Quantus Fluorometer (Promega, Madison, WI, USA). DNA libraries were prepared using the Nextera XT Prep Kit and pooled for paired-end sequencing of 300-bp reads with the Reagent Kit at 300 cycles into a Miseq platform (Illumina, Inc., San Diego, CA, USA). Fluorescent images were assessed using the MiSeq Control Software, and FASTQ-formatted sequence data was generated with the MiSeq Reporter Analysis Software. The density cluster and Q-score ≥ 30 of sequenced reads ranged, respectively, from 1185 to 1196 k/mm^3^ and from 85 to 88%, confirming the good quality of the sequencing runs. The DNA of one clinical *H. pylori* isolate (i.e., ID: DRC64) intended to be used as an internal control in bioinformatics was additionally supplied to the National Institute of Genetics (Tokyo, Japan) for sequencing using the Pacific Biosciences (PacBio) and the Illumina Hiseq platforms.

### 2.4. Bioinformatics

Genomic PacBio and Illumina Hiseq data obtained for the isolate DRC64 were assembled and polished into a single complete genome sequence by the National Institute of Genetics (Tokyo, Japan). The data obtained for each isolate using the Illumina Miseq platform were either exploited as high-throughput short reads or an assembled draft genome. The Genomics Workbench Software v. 8.5.1 (CLC bio, Aarhus, Denmark) was used to filter and pair short reads, assess the data quality, and trim low-quality bases (< Q30) and adapters. In further analyses, following the recommendations of Illumina, we selected samples with more than 80% reads at quality ≥ Q30. The draft WGS was de novo assembled from Illumina paired-end short reads with SPAdes v. 3.14.0 [22]. Then, after a quality assessment of the obtained draft WGS with QUAST v. 5.0.2 [23], gene sequences were identified and annotated using the RASTtk pipeline in the Rapid Annotation using the Subsystem Technology v. 2.0 [24]. The WGS-based method applied in this study was validated using the data obtained with the strain DRC64 through various methodological approaches in use for the discovery of putative mutations encoding AMR. For a more thorough description of the validation process, see Supplementary Materials, Appendix A. Plasmids with potentially encoding AMR-related features were systematically searched in high-throughput short reads by using the PlasmidSeeker tool [25] against the related reference database (http://bioinfo.ut.ee/plasmidseeker/; accessed on 17 December 2019) including 8514 plasmids of which 41 had been found in the *H. pylori* species. Nucleotide sequence datasets and inferred amino acid sequences were aligned and visually analyzed using the CLC genomic Workbench v. 8.5.1 and MEGA v. 7 [26]. The promoter region sequences likely involved in AMR were retrieved from WGS using the BLASTN against a local database created with draft WGS of clinical isolates in the CLC genomic Workbench v. 8.5.1. The three-dimensional structure model of proteins was constructed using the Swiss-Modeller server [27]. Putative AMR-encoding genes were defined based on previous reports [28,29]. Mutations were reported following standard recommendations in molecular diagnostics from the Human Genome Variation Society (HGVS) [30,31], as detailed in Supplementary Materials, Appendix B.

### 2.5. Statistical Analyses

Statistical analyses were performed using the R Software v. 3.5.3 (the R development Core Team, R Foundation for Statistical Computing, Vienna, Austria). Qualitative variables were compared using the Chi-squared test or Fisher’s exact test, as appropriate. The agreement between results from various methods was measured using the Cohen’s Kappa index with a related 95% confidence interval. The diagnostic performance of WGS for predicting phenotypic AST was evaluated using the Youden’s index, sensitivity, and specificity with a corresponding 95% confidence interval. For all statistical tests, we considered a *p*-value < 0.05 as statistically significant.

### 2.6. Nucleotide Sequence Accession Number

All nucleotide sequences analyzed in this study were deposited in the DNA Data Bank of Japan (DDBJ) under accession number ID: LC537338-LC537442.

### 2.7. Ethical Issues

All human subjects provided their informed consent for inclusion before participation in the study. The study was conducted in accordance with the Declaration of Helsinki, and the protocol was approved on 30 August 2016 by the National Ethics Committee of the Ministry of Public Health in DRC (Project N#032/CNES/BN/PMMF/2016).

## 3. Results

### 3.1. Baseline Characteristics of Patients Included in the Study

All patients included in this study were Congolese (with a mean age ± standard deviation of 45.3 ± 15.3 years old; 58% were females) and had not been previously treated for *H. pylori* eradication. One hundred and nine strains (49.5% of patients) were successfully isolated and assessed for phenotypic AST. Of these, three isolates were excluded from the analyses as their very slow growth did not allow for the accurate performance of AST. One hundred and two *H. pylori* isolates (93.5% of all strains), which provided high-quality WGS data, were included in the final analyses.

### 3.2. H. pylori Primary Antimicrobial Susceptibility in Kinshasa, DRC

**Figure 1:**
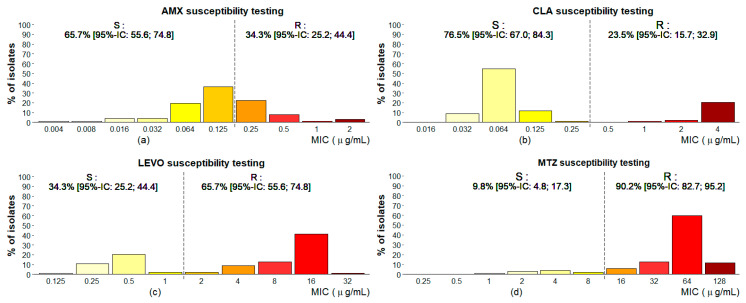
Antimicrobial susceptibility testing using the agar dilution method. This figure depicts the outcomes of antimicrobial susceptibility testing (AST) using the agar dilution method on 102 *H. pylori* strains that were collected from Kinshasa, DRC. Clinical breakpoints were defined following the guidelines of the European Committee on Antimicrobial Susceptibility Testing (EUCAST) and are shown as a black dashed line. Rates of resistant (R) and susceptible (S) isolates with 95% confidence intervals (95%-CI) are also indicated in panels (**a**–**d**) for amoxicillin (AMX), clarithromycin (CLA), levofloxacin (LEVO), and metronidazole (MTZ).

The antibiogram of 102 *H. pylori* strains from Kinshasa is shown in Figure 1 and summarized in Table 1. Resistance rates to MTZ, LEVO, AMX, and CLA were 90.2%, 65.7%, 34.3%, and 23.5%, respectively. Notably, 96.1% of strains (*n* = 96) were resistant to at least one of the antimicrobials tested, and 73.5% had a multidrug resistance (MDR). The most frequent MDR was a dual resistance to LEVO and MTZ (28.4% of strains).

### 3.3. Validation of the Method Used for Assessing AMR-Related Genetic Determinants

Four different WGS-based methods that are applicable for detecting AMR-related genetic determinants were compared (see Supplementary Materials-Appendix A for more details). Full-length genes retrieved from the de novo assembled draft WGS (Figures S1 and S2) showed the best congruence with gold standard sequences of all tested AMR-related genes (Kappa = 1.0; TP and TN rates of 100%; *p* < 0.0001) (Table S1). This method was applied in further analyses.

### 3.4. AMR-Related Genetic Determinants and Prediction of Phenotypic Resistance

A possible role of genes encoded by plasmids was ruled out since, while screening WGS reads with the PlasmidSeeker tool [25], we detected a no *k*-mer fraction related to any of the plasmids available in the reference database. This pointed towards the primary importance of determinants encoded by the bacterial genome, rather than plasmids, in mediating phenotypic AMR in our isolates.

#### 3.4.1. Amoxicillin Resistance (AMX-R)

At first, we suspected the role of β-lactamase production in the noted *H. pylori* AMX-R (34.3%) that frequently induces resistance to β-lactam antimicrobials in Gram-negative bacteria [32]. However, there was no β-lactamase activity detected by the chromogenic cephalosporin method in any of our *H. pylori* isolates, excluding the possible role of β-lactamase in AMX-R in this study. Then, we screened the WGS data of our strains to identify eventual structural alterations within the *pbp1A*, *pbp2*, *pbp3,* and *pbp4* genes that encode penicillin-binding proteins (PBPs). We focused on conserved penicillin-binding motifs (PBP-motifs), i.e., the SXXK, SXN, and KTG motifs, and specific codons that have been studied in relation to AMX-R in *H. pylori* [33]. Of these four genes, mutations that were statistically relevant for AMX-R (*p* < 0.05) could be observed only in the *pbp1A* gene, namely onto three PBP-motifs (SAIK_368_371_, SKN_402_404_, KTG_555_557_, and SNN_559_561_) and at three *C*-terminus codons (A474, T558, T593, and G595) in 29 out of 35 AMX-R strains, as shown in the details in Supplementary Materials, Table S2. Interestingly, these mutations included five new putative AMX-R encoding genotypes: F366L (one AMR-R), S405N (five AMX-R strains), A474T (one AMX-S vs. five AMX-R), T558S (five AMX-R), and N562H (one AMX-R) (Figure S3). Moreover, they also included mutations at five codons (S402G, S414R, T556S, N562Y, and T593A/G/K/S) that had been previously proven in AMX-R by natural transformation. Contrarily, mutations of STGK_338_341_ as well as variants such as V469A/M, N562D, and T599P/V seemed to be unrelated to AMX-R in our *H. pylori* strains. Otherwise, all mutations affecting PBP-motifs of *pbp2* and *pbp3* were not significantly linked to resistance and occurred dually with *pbp1A* mutations, likely suggesting the lack of an independent role in AMX-R. The only mutation found in PBP-motifs of the *pbp4* gene occurred in an AMX-S strain. Unexpectedly, six AMX-R strains did not show any alteration of the PBP-motif in the *pbp1A*, *pbp2*, *pbp3,* or *pbp4* gene, whereas one phenotypic AMX-S strain had a mutation linked to resistance in the *pbp1A* gene (S402G in the SKN_402_404_ motif). Finally, no mutation in the membrane proteins encoded by the *hofH*, *hefC*, or *hopC* genes that had been invoked in AMX-R could be detected. Overall, we noted an almost perfect agreement (Kappa = 0.842%; *p* < 0.0001) between phenotypic AST and relevant mutations compiled in the full-length *pbp1A* gene (Table 2). The genotypic AMX susceptibility testing based on *pbp1A* gene mutations showed a Youden’s index, sensitivity, and specificity of 0.8136%, 82.9%, and 98.5%, respectively, when phenotypic AST is set as the gold standard (Table 2).

#### 3.4.2. Clarithromycin Resistance (CLA-R)

Phenotypic CLA-R was significantly associated with an adenine to guanine substitution at nucleotide-positions A2142 (16.7% of CLA-R) and A2143 (70.8% of CLA-R) in domain V of the gene encoding 23S rRNA (*p* < 0.0001). A C2289T mutation detected in two isolates did not show any link to resistance (*p* = 0.417) (Table S3). Three CLA-R strains did not display any relevant mutation in the 23S rRNA sequence. A G160A mutation of *infB* (encoding a translation initiation factor, IF-2) that may induce CLA-R according to our previous study in Vietnam [34] was detected in one susceptible isolate, whereas putative mutations of the *rpl22* gene (encoding a ribosomal protein that interacts with the 23S rRNA domains) were not noted. Overall, genotyping alleles at codon-positions 2142 to 2144 in the sequence encoding domain V of the 23SrRNA showed an almost perfect agreement with the phenotypic CLA-R (Kappa = 0.91; *p* < 0.0001). Overall, the process showed a sensitivity of 87.5%, a specificity of 100.0%, and a Youden’s index of 0.875 (Table 2).

#### 3.4.3. Levofloxacin Resistance (LEVO-R)

Mutations at specific codons in and outside the quinolone resistance-determining region (QRDR) of single or dual genes encoding DNA gyrase subunit A and B (*gyrA* and *gyrB*), that have been previously associated with LEVO-R in *H. pylori* [35,36] could be identified in our study, as shown in Supplementary Materials, Table S4. We observed that the phenotypic LEVO-R was significantly associated with mutations in the QRDR (between A71 and Q110) encoded by the *gyrA* gene (*p* < 0.0001), notably those arising from codons N87 (*p* < 0.0001) and D91 (*p* = 0.0002) in contrast to codons A92 (*p* = 0.258) and R103 (*p* = 1.000). Mutations in the QRDR (E415 to S454) of *gyrB* were present at codons D435 and V437 in only two LEVO-R strains (3.0%; *p* = 0.545), including one strain with MIC = 4 µg/mL carrying D435N but no relevant mutation in the *gyrA* QRDR. Among the SNPs located outside QRDR and that had previously been suspected to induce LEVO-R, we detected R130K (11.9% of LEVO-R vs. 34.3% of LEVO-S strains; *p* = 0.0095) and A129T (one LEVO-R strain) in *gyrA* and R484K (one LEVO-S strain) in *gyrB*. Noteworthy, the *gyrA* gene showed a new variant among mutations in QRDR: A92T (carried by one LEVO-S vs. seven LEVO-R isolates). In addition, we suspected two new mutations of contributing to LEVO-R, A129T in *gyrA* (one LEVO-R strain), and R579C in *gyrB* (one LEVO-S vs. seven LEVO-R strains) (Figure S3). Unexpectedly, three phenotypically susceptible isolates (8.6%) had mutations in the QRDR sequence of *gyrA* while three other strains that were phenotypically LEVO-R (at MIC = 4 µg/mL) showed no relevant mutation in both *gyrA* and *gyrB*. The congruence between genotypic and phenotypic AST was almost perfect when considering mutations of the QRDR sequence in both *gyrA* and *gyrB* (Kappa = 0.828; *p* < 0.0001). Compiling mutations carried in the QRDR of these genes for predicting phenotypic AST showed a sensitivity of 92.5%, a specificity of 91.4%, and a Youden’s index of 0.839 (Table 2).

#### 3.4.4. Resistance to Metronidazole (MTZ-R)

Various mutational changes affecting several genes (e.g., *rdxA*, *frxA*, *recA*, *sodB*, *fur, mdaB, ribF*, *omp11*, and *rpsU*) that encode putative MTZ-reducing enzymes, DNA repair proteins, proteins regulating cell responses to oxidative stress, and possible outer membrane proteins have been shown or hypothesized as being involved in the development of MTZ-R in *H. pylori* [37,38,39,40,41,42,43,44,45,46,47]. Thus, we conducted an extended structural analysis of full-length genes encoding these proteins in the hope of assessing their respective clinical relevance. The results observed are presented in Supplementary Materials, Table S5A,B. Overall, mutations related to resistance could be identified mainly in genes encoding the oxygen-insensitive NAD(P)H nitroreductase (*rdxA*) and the NAD(P)H flavin nitroreductase (*frxA*) and secondarily in genes encoding the ferric uptake regulator (*fur*) and in the promoter region of superoxide dismutase (*sodB*). In the *rdxA* gene, null mutations were noted exclusively in MTZ-R strains (*p* = 0.0014) and included frameshift mutations (*n* = 25), premature stop codons (*n* = 12), and, interestingly, sequence deletions (*n* = 10) and insertions (*n* = 1). We could identify five types of variable sequence deletions in the *rdxA* sequence, K2_M21del, S92_Q146del, R131_K166del, K168_V172del, (N178_L185del; G189_R200del), L137_I142del, and K2_M21del, leading to a predictable loss of binding sites for molecules of flavin mononucleotide (FMN) cofactor and likely a loss or decrease of the MTZ-reducing activity (Figure S4). We identified additional complex variants of *rdxA* mutations, including a large inserted sequence (one isolate with K168_V169insSGRDFRTAYQTer) and a no-stop mutational change (one isolate with Ter2011LextTer25). However, unlike the inserted sequence ending with a stop codon, the no-stop mutational change, which is a mutation affecting the translation termination codon by introducing a new downstream termination codon that extends the C-terminus of the protein, did not show any predictable alteration of the protein function. Point-mutations at functional codons were also detected in *H. pylori* strains that had a conserved *rdxA* gene, including 30.4% of MTZ-R and 10% of MTZ-S strains (*p* = 0.274). Globally, null mutations and substitutions at functional codons were identified in 72.8% of MTZ-R strains (versus 10% of MTZ-S strains; *p* = 0.0026). In the *frxA* gene, null mutations included frameshift mutations (*n* = 23) and premature stop codons (*n* = 15). In addition, we noted point-mutations carried at functional codons (*n* = 3). A dual inactivation by null mutations in proteins encoded by *rdxA* and *frxA* genes was noted in 25 MTZ-R isolates (27.2% of MTZ-R) while the nitroreductase encoded by *frxA* was additionally noted in four MTZ-S strains (40% of MTZ-S). Five MTZ-R strains harbored no known functional mutation in *rdxA* and/or *frxA* genes (5.4% of MTZ-R; *n* = 5), likely suggesting additional resistant mechanisms. Two MTZ-R strains otherwise with an inactivated *rdxA* gene were found carrying two mutations previously related to resistance, an A-5C single-base pair substitution in the *sodB* promoter region and a P114S codon substitution in the *fur* gene. No mutation previously related to the phenotypic MTZ-R could be observed in the *recA* gene encoding a protein involved in the DNA recombination and repair (i.e., Y103H and S121D), in *mdaB* encoding the modulator of drug activity (i.e., R99I and G98D), in the *ribF* gene of riboflavin (i.e., T222M and A227T), in the *omp11* gene of outer membrane protein 11 (i.e., A1290D), and in the *rpsU* gene of 30S ribosomal protein S21 (i.e., D13T). With the exception of *rdxA* and *frxA* genes that could encode null mutations, other explored genes were well-conserved and were in the wild-type at variable frequencies regardless of the AST phenotype of the strains (Table S5B). The presence of a wild-type sequence was statistically associated with a phenotypic MTZ susceptibility only in *rdxA* (*p* = 0.003). Overall, compiling functional mutations across the *rdxA* sequence for predicting the phenotypic MTZ-R showed a sensitivity, specificity, and Youden’s index of 72.8%, 90.0%, and 0.625%, respectively. A reasonable agreement (Kappa = 0.304) was noted between the results of genotypic and phenotypic AST (Table 2).

## 4. Discussion

This study was conducted to assess the rate of phenotypic AMR, identify related genetic determinants, and explore the feasibility of genomic NGS-based approaches for tracking resistance in *H. pylori* clinical isolates from the DRC. Therefore, we first assessed the susceptibility of *H. pylori* strains to AMX, CLA, LEVO, and MTZ, which are critical antimicrobials conventionally used in first- and second-line *H. pylori* eradication therapy, in combination with proton pump inhibitors (PPIs) [6]. The magnitude of *H. pylori* resistance to the four drugs was 33.0%, 22.6%, 90.6%, and 66.9%, respectively. For the first time in three decades, this study has provided information on the drug susceptibility of *H. pylori* in the DRC. A unique report on single drug susceptibility was released in 1990 recording 84% of MTZ-R within a rural area located in the Eastern part of the country [48]. The profile of resistance noted in this study is globally consistent with observations in several other African countries that have been compiled in a recent meta-analysis [49]. In the DRC, as in other African countries [17], there are no official guidelines addressing the *H. pylori* treatment based on local epidemiology. Given the thresholds of resistance rates put forward by the Maastricht V Consensus Report [6], our outcomes suggest that, ideally, AST should be performed ahead of treating *H. pylori* and empirical treatments based on all four drugs should be abandoned in a clinical practice. Generally, high resistance rates correlate with a persistent selection of mutants in a country owing to the frequent use of drugs [50]. In the DRC, in particular, self-medication or the prescription of these drugs for several other infections (e.g., typhoid fever, upper respiratory tract infections, amoebiasis, giardiasis) is very common in the population. Therefore, public health policy makers should take steps to stem the emergence and spread of the antibiotic resistance of *H. pylori* and control the use of antimicrobials in the country. Additional research is needed, including assessing the efficiency of alternative *H. pylori* eradication regimens, to strengthen the local clinical practice.

To identify possible AMR-driving molecular mechanisms, we explored a massive Illumina-based WGS data. As we could not detect any plasmid in our isolates, we ensured that all resistance determinants were encoded in the bacterial genome. A WGS-based approach validated for the discovery of variants in AMR-related genes was applied to screen targeted genes in each isolate. We observed that the phenotypic AMX-R could mainly be related to mutations altering the *pbp1A* gene at PBP-motifs (i.e., SAIK_368_371_, KTG_555_557_, and SNN_559_561_) and at c-terminus codons (i.e., T593 and G595). Hence, the mutations found in our isolates correspond to previous studies based on site-directed mutagenesis using non-African *H. pylori* populations [51,52,53]. Interestingly, we additionally detected a set of putative new mutations probably encoding AMX-R (F366L, S405N, T558S, and N562H) that warrant further exploration in vitro. Therefore, AMX-R in our strains could be due to a reduced affinity for the protein encoded by the *pbp1A* gene. An independent role played by a few mutations noted in PBP-motifs of *pbp2*, *pbp3*, and *pbp4* genes could not be evidenced in AMX-R. This is consistent with the result reported by Rimbara E et al. that mutations in other *pbp* genes might also be related to AMX-R in *H. pylori* but by facilitating and enouncing the primary resistance owing to mutations in the *pbp1A* gene [54]. The CLA-R of our *H. pylori* clinical isolates emerged from two types of point-mutations, A2142G and A2143G, in the domain V of the 23S rRNA gene. The vast majority of published studies have confirmed that these positions in the peptidyl transferase encoding region of the 23S rRNA gene are the most prevalent mutations and account for nearly 90% of primary CLA-R in Western countries [28,29]. The predominance of A2143G in MTZ-R (70.8%), congruent with the outcome from a meta-analysis compiling CLA mutations in Africa [49], raise concerns since this mutation (as compared to A2142C or 2142G) is thought to be mainly responsible for failure during an attempted *H. pylori* eradication with the CLA-based therapy [29]. No additional change previously linked to CLA-R could be observed outside the domain V of the 23S rRNA gene as well as in other targeted genes, *rpl22* and *infB* [28,34]. The LEVO-R observed in our study could be attributed primarily to mutations of the QRDR encoded by *gyrA* and *gyrB* genes. In the QRDR of *gyrA,* the mutations found were located at codons 87 (N to K, I, T) and 91 (D to Y, G, H) in 91% of resistant strains. This result globally converges with previous observations establishing these two codons as hotspots for LEVO-R [9,11,28,55]. However, in addition, a new codon of the QRDR of *gyrA* was also triggered by a mutation (i.e., A92T) for which the relevance for LEVO-R still needs to be fully assessed in future studies. Moreover, we detected the mutation R130K, located outside the QRDR of *gyrA*, that had previously been suspected in LEVO-R elsewhere [56]. However, this mutation was frequently noted in both resistant and susceptible strains and appeared to be unrelated to resistance in our study.

Tracking MTZ-R-encoding genotypes in *H. pylori* is challenging since an impressive set of mutational changes altering several genes may be involved [41,43,44,47,57,58]. Therefore, we explored the massive sequence information resulting from genes that encode putative MTZ-reducing electron acceptors (15 genes), enzymes involved in DNA repair and in response to oxidative stress (four genes), and additional proteins recently invoked in MTZ-R (four genes). Various genotypes that had been reported as conferring MTZ-R were mainly detected in *rdxA* and *frxA* genes, and rarely in the *fur* gene and in the promoter region of the *sodB* gene. MTZ-R-related genotypes in *rdxA* and/or *frxA* included null mutations and point-mutations at functional codons according to the most updated classification of MTZ-R-related mutations by Martínez-Júlvez et al. [59]. None of these MTZ-R genotypes encoded by *rdxA* could be noted among susceptible isolates. Notably, while analyzing the *rdxA* gene, we realized that WGS could resolve complex variants of mutations that would be challenging to detect with classical PCR-based methods. These variants included deletions of intercalary sequence (*rdxA*-dels in 10% of MTZ-R isolates), large inserted sequences ending by a stop codon (one MTZ-R isolate), and no-stop mutational changes (one MTZ isolate). Additional analyses based on the protein structure predicted that *rdxA*-dels were inactivating the *rdxA* gene in clinical isolates as the resulting proteins were structurally unable to bind FMN molecules, the cofactor essential for the MTZ-reducing enzymatic activity [59]. Further evidence for the *rdxA-*dels as MTZ-R encoding genotypes was obtained by complementation of genes with a *rdxA*-del from MTZ-R strains to MTZ-S strains that induced resistance (our unpublished data). In contrast to *rdxA*, the role of other putative MTZ-R-related genes was less evident. In summary, mutations in the *rdxA* gene appeared to be the main factor driving MTZ-R in our isolates, as a wild-type gene was significantly associated with MTZ-S, while possible functional mutations of *rdxA* correlated with MTZ-R. MTZ-R in strains that harbored no *rdxA* and/or *frxA* mutations (5.4% of MTZ-R in our study) had been suggested to result from a down regulation of *rdxA* expression by putative mutations falling in the promoter region of *rdxA* [60,61]. To assess this hypothesis, however, the promoter region of the *rdxA* gene still needs to be identified and characterized in future studies [61].

To assess the ability of the WGS-based approach for predicting the phenotypic AST, we detected and compiled individual mutations altering functional sites in sequences showing a plausible link with resistance to each assessed drug. Therefore, we found that the WGS information had a high performance in predicting phenotypic AST to AMX (i.e., based on the full-length *pbp1A* gene), CLA (i.e., based on domain V of the 23S rRNA gene), and LEVO (i.e., based on QRDRs of the *gyrA* and *gyrB* genes) and moderate performance in predicting MTZ susceptibility (full-length *rdxA* gene). This converges with recent observations from strain populations collected in Europe and Asia [9,11]. Our results demonstrate that WGS can thus be applied as an accurate alternative of the phenotypic AST in an African *H. pylori* genetic population. WGS-based methods present several advantages over traditional molecular-based methods, such as those based on the polymerase chain reaction (PCR), that are often used to detect AMR genotypes. For instance, when AMR may arise from scattered sequence positions (e.g., in genes such as *pbp1A* and *rdxA*), it is crucial that the molecular-based approach covers a sufficient sequence length to reach its highest performance in detecting an encoded resistance. In contrast, PCR-based methods would face limitations since they can accurately target only a limited number of nucleotides and hence, would be prone to false-negative results. In addition, unlike WGS-based approaches, traditional methods would not be able to cover all possible complex structural variants of AMR genotypes (e.g., large deletions or insertions in *rdxA*).

This study referred to culture-based phenotypes as the gold standard for AST. This would be justified as an antimicrobial therapy is consensually based on in vitro phenotypic susceptibly information [6]. However, it is important to bear in mind that the phenotypic AST in *H. pylori* has important limitations. First, there are challenges owing to the pathogen’s fastidious growth requirements, restricting the phenotypic AST assay to only well-equipped laboratories with well-trained technicians. For the three strains included in our study, the phenotypic AST could not be performed because of their extremely low growth. Second, interpretation of phenotypic outcomes may frequently be difficult and non-reproducible in *H. pylori*. This is the case, for instance, when performing MTZ susceptibility testing that can potentially be affected by redox variations in the test medium [9]. Finally, assessing the phenotype of AMR in *H. pylori* is time-consuming and delivers results in about two weeks in the best cases [8]. Therefore, it is particularly challenging to implement the procedure in a clinical practice, especially in situations with a high *H. pylori* prevalence. All these factors combined prevent regions with limited resources, such as most African countries, from performing the routine phenotypic AST of *H. pylori* [17]. In contrast, bacterial WGS by NGS can be performed from the initial bacterial culture with a reproducible outcome and within an acceptable timeframe (≤ 72 h) [62]. The relevance of the WGS outcome can be easily assessed by the retrospective analysis of sequence data, whereas the phenotypic AST would need to be retested using stocked *H. pylori* samples that often have a decreased ability to grow in vitro [9]. Moreover, in recent years, NGS technologies and WGS data analytical methods have become increasingly more available and cost-effective (e.g., using Illumina Miseq or Oxford Nanopore technologies). They can probably now be afforded even by laboratories located in low-income regions. For all the aforementioned reasons, exploiting bacterial genomics for tracking the AMR of *H. pylori* is an attractive option in African countries compared to the phenotypic AST and AMR genotyping by classical PCR-based molecular methods.

This study had some limitations. The results observed in this study might not be extrapolated to other populations in Africa as only a single city was used for the collection of samples. The ability of using WGS for predicting a phenotypic AST can be hampered by hetero-resistance when mixed *H. pylori* populations of the resistant and susceptible strains exist in the same patient [8,63,64]. Furthermore, it is likely that new mechanisms conferring AMR had arisen but could not be covered by this assay (e.g., in resistant isolates with no relevant mutation of *23SrRNA*, *pbp1A*, *gyrA/gyrB*, and *rdxA/frxA*). Hence, both putative hetero-resistance and yet unknown mechanisms of resistance would partially explain the lack of congruence between outcomes from phenotypic and genotypic AST that occurred in some isolates in this study. AMR-related mutations were frequently underrepresented, making it difficult to reach statistical significance when assessing single genotypes. Since the emergence of drug resistance seems to be multifactorial in *H. pylori*, our study could not definitively rule out the incremental contribution of multiple genes or genetic loci to the level of resistance for which primary mechanisms were identified in this study. In this study, we did not explore the potential of intrinsic efflux mechanisms for single or multidrug resistance as already demonstrated in *H. pylori* elsewhere [65,66,67,68,69]. This would require further analyses such as transcriptional analyses, efflux pump inhibition assays, drug accumulation experiments, and generation of mutants (e.g., knocking out the *hefABC*, *hefDEF*, and *hefGHI* genes), which were beyond the scope of our study. Otherwise, we realized that the WGS assembly obtained from the Illumina Miseq short reads provided non-redundant datasets restricting the analyses of possible duplicated AMR-related genes (i.e., the *23Sr RNA* genes) to only one sequence copy. We believe that other NGS technologies providing long read data (e.g., Oxford Nanopore, Illumina Hiseq, or PacBio) are better equipped to address this limitation. Thus, the approach outlined in this study could be enhanced by further development and research.

## 5. Conclusions

In summary, this study found that increased rates of AMR exist in the DRC and require susceptibility testing-guided therapies instead of empirical treatments, to appropriately counteract *H. pylori* infections. Mutations that are clinically relevant for predicting most drug resistance in the study population could be identified, including mutations previously reported in Western and Asian countries but also several newly described mutations likely encoding not yet reported resistance mechanisms. Moreover, this study shows that WGS-based methods are feasible alternatives to conventional phenotypic AST in *H. pylori*. In particular, the WGS approach raised in this study, using draft genomes instead of raw high throughput reads mapped to the reference, shows a higher accuracy compared to approaches frequently used. This study further reinforces the acknowledged usefulness of molecular methods for detecting and evaluation microorganisms (e.g., viruses, bacteria, protozoa) in clinical samples, without the requirement of advanced microbiological processes (e.g., isolation by cultures, phenotypic AST) that are often time-consuming and technically demanding [11,62,70,71]. In the DRC, specifically, enhanced efforts are needed locally to control antimicrobial use, tackle the emergence and spread of bacterial resistance, and explore the clinical efficiency of applicable regimens (e.g., bismuth-containing regimens, AST-directed regimens, newer drugs, and drug adjuvants). Implementing WGS for tracking AMR in the country would be very informative for clinical decisions, surveillance activities, and research targeting the underlying molecular mechanisms.

## Figures and Tables

**Table 1:** Profile of the phenotypic antimicrobial resistance (AMR) of *H. pylori* clinical isolates from Kinshasa, DRC.

Phenotypic Susceptibility Profile	*n*	% (95%-CI)
Susceptibility to all antimicrobials	5	4.9 (2.1; 10.9)
Resistance to at least one antimicrobial	98	96.1 (90.3; 98.5)
MTZ-R	92	90.2 (82.9; 94.6)
LEVO-R	67	65.7 (56.1; 74.2)
AMX-R	35	34.3 (25.8; 43.9)
CLA-R	24	23.5 (16.4; 32.6)
Single drug resistance	21	20.6 (13.9; 29.4)
MTZ-R only	17	16.7 (10.7; 25.1)
CLA-R only	2	2.0 (0.3; 6.9)
LEVO-R only	2	2.0 (0.3; 6.9)
AMX-R only	1	1.0 (0.1; 5.3)
Multidrug resistance	75	73.5 (64.2; 81.1)
LEVO-R + MTZ-R	29	28.4 (20.6; 37.8)
AMX-R + LEVO-R + MTZ-R	15	14.7 (9.1; 22.9)
CLA-R + LEVO-R + MTZ-R	10	9.8 (5.4; 17.1)
AMX-R + CLA-R + LEVO-R + MTZ-R	8	7.8 (4.0; 14.7)
AMX-R + MTZ-R	5	4.9 (2.1; 10.9)
CLA-R + MTZ-R	3	2.9 (0.8; 8.3)
AMX-R + LEVO-R	2	2.0 (0.3; 6.9)
CLA-R + LEVO-R	2	2.0 (0.3; 6.9)
AMX-R + CLA-R + MTZ-R	1	1.0 (0.1; 5.3)

AMX: Amoxicillin; CLA: Clarithromycin; LEVO: Levofloxacin; MTZ: Metronidazole; R: Resistant; and S: Susceptible.

**Table 2:** Performance of whole genome sequencing (WGS)-based AST using next-generation sequencing (NGS).

Genotypic AST (Antimicrobial)**	Phenotypic AST				*p*-Value	Se	Sp	Youden’s Index (95%-CI)	Cohen’s Kappa (95%-CI)
	Resistant		Susceptible						
	*n*	%	*n*	%					
*pbp1A* gene mutations (AMX)									
Resistant	29	82.9	1	1.5	<0.001	82.9	98.5	0.8134 (0.583; 0.934)	0.842 (0.729; 0.955)
Susceptible	6	17.1	66	98.5					
Domain V of *23S rRNA* gene (CLA)									
Resistant	21	87.5	0	0.0	<0.001	87.5	100.0	0.875 (0.630; 0.973)	0.914 (0.819; 1.000)
Susceptible	3	12.5	78	100.0					
QRDR of *gyrA* and *gyrB* genes (LEVO)									
Resistant	62	92.5	3	8.6	<0.001	92.5	91.4	0.839 (0.604;0.957)	0.828 (0.714;0.942)
Susceptible	5	7.5	32	91.4					
*rdxA* gene (MTZ)									
Resistant	59	64.1	0	0.0	<0.001	64.1	100.0	0.641 (0.226;0.739)	0.304 (0.073;0.535)
Susceptible	33	35.9	10	100.0					

Genotypes that were considered included (See Supplementary Materials—Appendix B for details): Alleles onto three PBP-motifs (SAIK_368_371_, SKN_402_404_, KTG_555_557_, and SNN_559_561_) and at three C-terminus codons (A474, T558, T593, and G595) encoded by the *pbp1A* gene for AMX AST; alleles at codon-positions 2142 to 2144 in the domain V of 23S rRNA encoding gene for CLA AST; alleles of the quinolone resistance-determining region (QRDR) of DNA gyrase A and B subunits encoded by *gyrA* and *gyrB* genes; and alleles altering functional sites and flavin mononucleotide (FMN) cofactor binding sites for MTZ AST. (**) Abbreviations are as following: AMX: Amoxicillin; CLA: Clarithromycin; LEVO: Levofloxacin; MTZ: Metronidazole; R: Resistant; and S: Susceptible; Se: Sensitivity; Sp: Specificity.

## Supplementary Materials

The following are available online at https://www.mdpi.com/2076-2607/8/6/887/s1, Appendix A: Validation of WGS-based methods for the detection of variants in AMR-related genes; Appendix B: Discovery of relevant mutations in AMR-related genes.
