# Supplementary material for: Next-Generation Sequencing of the Whole Bacterial Genome for Tracking Molecular Insight into the Broad-Spectrum Antimicrobial Resistance of Helicobacter pylori Clinical Isolates from the Democratic Republic of Congo
Source: Microorganisms. 2020 Jun 11;8(6):887. doi: 10.3390/microorganisms8060887 (PMC7356661; doi:10.3390/microorganisms8060887)
Supplement: Supplementary file 1 [file microorganisms-08-00887-s001.pdf]

Additional file - Supplementary Materials

Next-generation sequencing of the whole bacterial genome for tracking molecular insight into the broad-spectrum antimicrobial resistance of *Helicobacter pylori* clinical isolates from the Democratic Republic of Congo

Evariste Tshibangu-Kabamba, Patrick De Jesus Ngoma-Kisoko, Vo Phuoc Tuan, Takashi Matsumoto, Junko Akada, Yasutoshi Kido, Antoine Tshimpi-Wola, Pascal Tshiamala-Kashala, Steve Ahuka-Mundeke, Dieudonné Mumba-Ngoyi, Ghislain Disashi-Tumba, and Yoshio Yamaoka*

(*) Correspondence: yyamaoka@oita-u.ac.jp; Tel.: +81-97-586-5740; Fax: +81-97-586-5749
Department of Environmental and Preventive Medicine
Oita University Faculty of Medicine
Oita, Japan

Contents

Appendix A. Validation process of the WGS-based method……………………………………………………2
Figure S1. Comparison of WGS-based methods for detecting AMR related genetic determinants in clinical isolates 3
Figure S2. Comparison of different WGS-based methods for the detection of variant in rdxA gene 4
Appendix B. Mutations detected in putative AMR-encoding genes of *H. pylori* clinical isolates from DRC 7
Table S2. Potential genotypes of AMX-R encoded in *H. pylori* clinical isolates from DRC..........................7
Table S3. Potential genotypes encoding CLA-R in *H. pylori* clinical isolates from DRC 10
Table S4. Potential genotype encoding LEVO-R in *H. pylori* clinical isolates from DRC 10
Table S5 (A). Potential genotype encoding MTZ-R in *H. pylori* clinical isolates from DRC.......................11
Figure S3. Protein structure of the oxygen-insensitive NAD(P)H nitroreductase encoded by rdxA gene 13
Table S6 (B). Potential genotype encoding MTZ-R in *H. pylori* clinical isolates from DRC 14
References 15

Appendix A. Validation process of the WGS-based methods

For the validation of the WGS methodological approaches in use for the discovery of AMR genetic determinants, we compared the performance of four different workflows applicable to NGS high-throughput data. The sequences from the complete WGS DRC64 obtained by combining PacBio and Illumina Hiseq long reads were used as a gold standard for the comparison. The reference sequences were retrieved from the complete WGS of 26695 (NCBI Reference Sequence: NC_000915.1). The tested outcomes were obtained with short reads from the Illumina Miseq data of the isolate DRC64 (both as an assembled draft WGS and as high-throughput short reads mapped to reference sequences). Different gene sequences were therefore compared for the detection of variants in putative AMR-related genes. As shown in **Figure S1**, the methods that were compared included: (i) variant detection using the consensus sequence from Illumina Miseq short reads of DRC64 mapped against reference genes from 26695 (Method 1); (ii) variant detection based on the full-length gene sequence retrieved from the de novo assembled and annotated draft WGS of DRC64 (Method 2); (iii) variants called directly from short reads mapped against reference gene sequences at a frequency ≥80% (Method 3) and (iv) at a frequency ≥0% (Method 4). In the first method, the consensus sequences were inferred using the NGS core tool of CLC Genomics Workbench v8.5.1. In the second method, annotated full-length genes of interest were retrieved from genomes and compiled using a customized script built with R Software v3.5.3 (The R development Core Team, R Foundation for Statistical Computing, Vienna, Austria). In methods 3 and 4, nucleotide variants were identified with probabilistic variant detection modules of CLC Genomics Workbench v8.5.1 using default parameters, variant detection set to 1, and reads with minimum coverage of 100.
The results obtained in different AMR-related genes are shown in **Table S1**. Calling variants directly from high-throughput short reads mapped to a reference sequence showed variable outcomes depending on the sequence assessed. The highest rates of falsely negative (22.5%) and falsely positive variants (54.5%) were noted when calling variants at 0% frequency from short reads mapped to *23S rRNA* and *pbp1A* genes, respectively. Relying on consensus sequences built from short reads mapped to a reference showed high performance for detecting variants, though falsely negative and positive variants were observed in the loci of *rdxA* (0.6% and 1.4%) and *gyrA* genes (0.1 and 1.8%). Falsely positive negative variants noted in the *rdxA* gene included ambiguous variants arising onto three loci as shown in **Figure S2**. Using full-length genes retrieved from de novo assembled draft WGS showed the best congruence with gold standard sequences of all tested AMR-related genes (Kappa = 1.0; TP and TN rates of 100%; $p < 0.0001$).

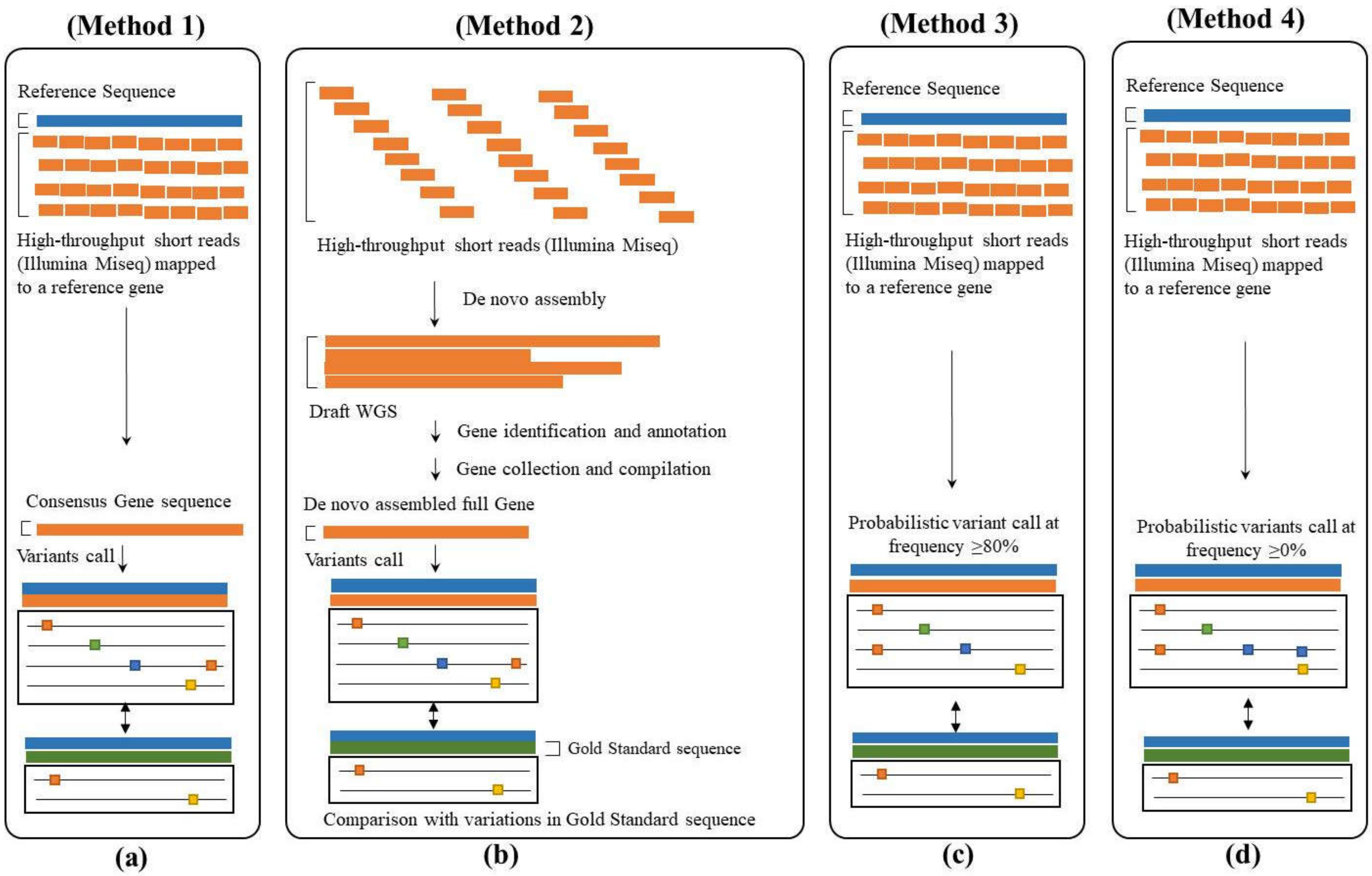

Figure S1. Comparison of WGS-based methods for detecting AMR-related genetic determinants in clinical isolates

This figure illustrates the different WGS-based methods that were compared for the detection of allelic variants in AMR-related genes. In panel (a), method 1 detects variants using the consensus sequence from the Illumina Miseq short reads of DRC64 mapped against a reference gene from the WGS of the *H. pylori* 26695 isolate. In panel (b), method 2 detects variants based on the full-length gene sequence retrieved from the de novo assembled and annotated draft WGS of DRC64. Method 3 and 4, in panels (c) and (d), call variants directly from short reads of DRC64 mapped against a reference gene sequence from 26695 at a frequency ≥80% (Method 3) and at frequency ≥0% (Method 4), respectively.

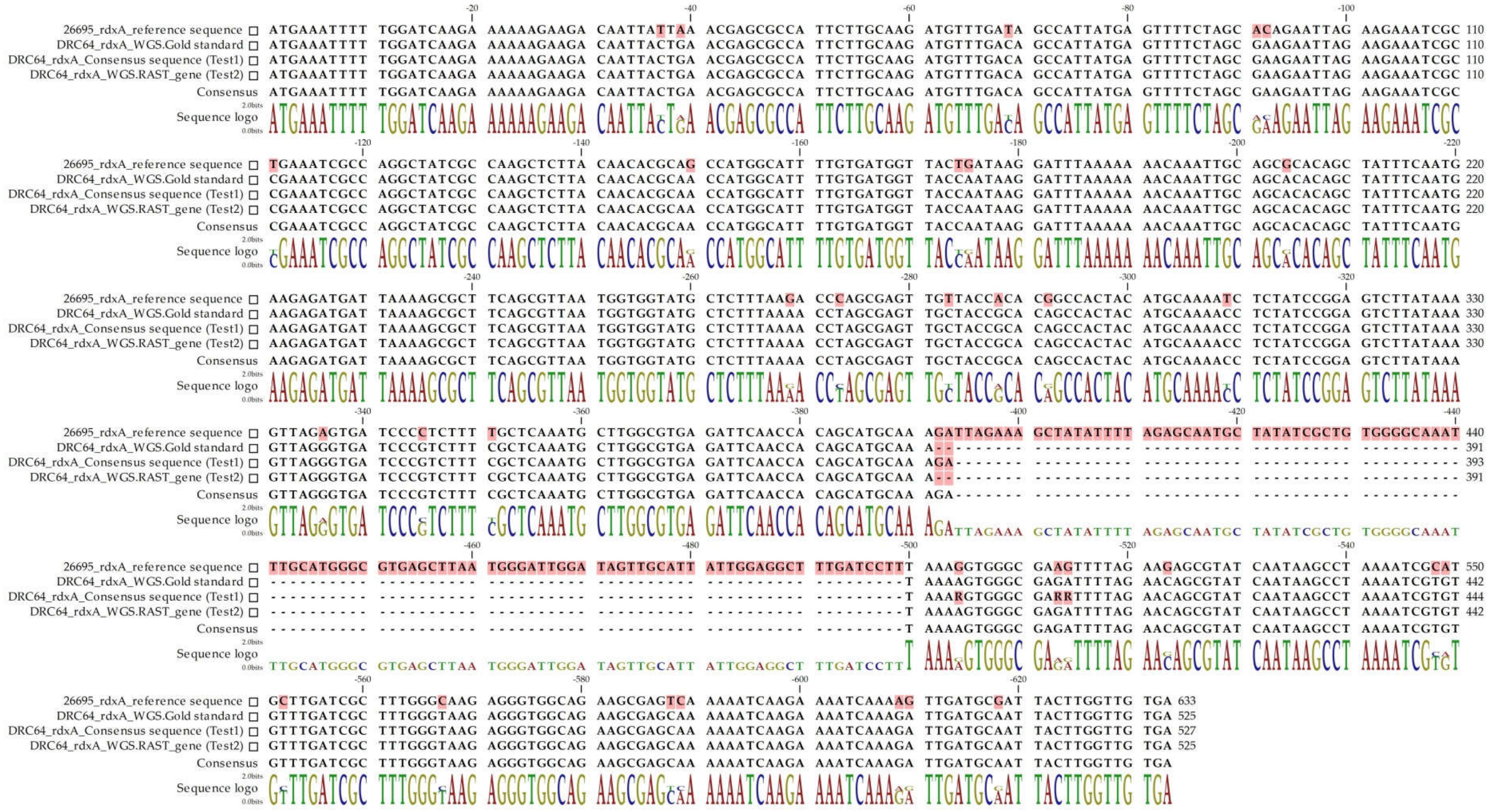

Figure S2. Comparison of different WGS-based methods for the detection of variant in the *rdxA* gene

This figure shows the sequence alignment of *rdxA* genes from *H. pylori* isolate 26695 and from *H. pylori* DRC64 obtained with the Classical Sequence Analysis tool of CLC genomic Workbench v8.5.1. From top to bottom, sequences are shown in the alignment as follows: *rdxA* reference gene from complete WGS of 26695, *rdxA* gold standard sequence from complete WGS of DRC64, *rdxA* test sequence 1 inferred from a consensus sequence built on the Illumina Miseq short reads of DRC64 mapped to the reference gene, and *rdxA* test sequence 2 retrieved from the draft WGS of DRC64 that was obtained with the Illumina Miseq short reads. The sequence logo calculated based on the alignment is also shown. Loci displaying allelic variations are shaded in red. Single-letter abbreviations for the nucleotide residues are as follows: A, Adenine; C, Cytosine; T, Thymine; and G, Guanine. The hyphen (-) indicates a gap noted in comparison with the nucleotide residues in the reference sequence. All other symbols in the sequence (e.g., R in *rdxA* test sequence 1) indicate ambiguous variants that could not be inferred by the assay.

Table S1. Performance of different WGS-based methods for detecting variants on AMR-related genes

AMR gene & applied WGS-based method	Performance*								Cohen's Kappa [95%-CI]	p-value
	TN		FN		FP		TP			
	n	%	n	%	n	%	n	%		
Penicillin-binding protein 1A gene (*pbp1A*)										
Variants detected in the consensus sequence	1898	100.0	0	0.0	0	0.0	88	100.0	1.000 [1.000; 1.000]	<0.001
Variants detected in the gene retrieved from draft WGS	1898	100.0	0	0.0	0	0.0	88	100.0	1.000 [1.000; 1.000]	<0.001
Variants called from mapped short reads. at 80% frequency	1729	91.1	169	8.9	54	61.4	34	38.6	0.183 [0.082; 0.284]	<0.001
Variants called from mapped short reads. at 0% frequency	1702	89.7	196	10.3	48	54.5	40	45.5	0.195 [0.100; 0.290]	<0.001
Large subunit ribosomal RNA gene (*23SrRNA*)										
Variants detected in the consensus sequence	2631	100.0	0	0.0	0	0.0	15	100.0	1.000 [1.000; 1.000]	<0.001
Variants detected in the gene retrieved from draft WGS	2631	100.0	0	0.0	0	0.0	15	100.0	1.000 [1.000; 1.000]	<0.001
Variants called from mapped short reads. at 80% frequency	2618	99.5	13	0.5	8	53.3	7	46.7	0.396 [0.139; 0.653]	<0.001
Variants called from mapped short reads. at 0% frequency	2039	77.5	592	22.5	5	33.3	10	66.7	0.022 [-0.047; 0.091]	<0.001
Gyrase subunit A gene (*gyrA*)										
Variants detected in the consensus sequence	2373	99.9	2	0.1	2	1.8	110	98.2	0.981 [0.963; 1000]	<0.001
Variants detected in the gene retrieved from draft WGS	2375	100.0	0	0.0	0	0.0	112	100.0	1.000 [1.000; 1.000]	<0.001
Variants called from mapped short reads. at 80% frequency	2373	99.9	2	0.1	11	9.8	101	90.2	0.937 [0.903; 0.971]	<0.001
Variants called from mapped short reads. at 0% frequency	2008	84.5	367	15.5	10	8.9	102	91.1	0.300 [0.235; 0.365]	<0.001
Gyrase subunit B (*gyrB*)										
Variants detected in the consensus sequence	2222	100.0	0	0.0	0	0.0	100	100.0	1.000 [1.000; 1.000]	<0.001
Variants detected in the gene retrieved from draft WGS	2222	100.0	0	0.0	0	0.0	100	100.0	1.000 [1.000; 1.000]	<0.001
Variants called from mapped short reads. at 80% frequency	2222	100.0	0	0.0	0	0.0	100	100.0	1.000 [1.000; 1.000]	<0.001
Variants called from mapped short reads. at 0% frequency	2015	90.7	207	9.3	13	13.0	87	87.0	0.403 [0.328; 0.478]	<0.001
Small subunit ribosomal RNA gene (*16SrRNA*)										
Variants detected in the consensus sequence	1485	100.0	0	0.0	0	0.0	17	100.0	1.000 [1.000; 1.000]	<0.001
Variants detected in the gene retrieved from draft WGS	1485	100.0	0	0.0	0	0.0	17	100.0	1.000 [1.000; 1.000]	<0.001
Variants called from mapped short reads. at 80% frequency	1483	99.9	2	0.1	4	23.5	13	76.5	0.810 [0.659; 0.962]	<0.001
Variants called from mapped short reads. at 0% frequency	1427	96.1	58	3.9	2	11.8	15	88.2	0.321 [0.152; 0.489]	<0.001

Oxygen-insensitive NAD(P)H nitroreductase gene (*rdxA*)										
Variants detected in the consensus sequence	491	99.4	3	0.6	2	1.4	137	98.6	0.977 [0.957; 0.997]	<0.001
Variants detected in the gene retrieved from draft WGS	493	100.0	0	0.0	0	0.0	140	100.0	1.000 [1.000; 1.000]	<0.001
Variants called from mapped short reads. at 80% frequency	493	82.0	108	18.0	0	0.0	32	100.0	0.316 [0.198; 0.433]	<0.001
Variants called from mapped short reads. at 0% frequency	488	81.9	108	18.1	5	13.5	32	86.5	0.297 [0.179; 0.414]	<0.001
NAD(P)H flavin nitroreductase gene (*frxA*)										
Variants detected in the consensus sequence	618	100.0	0	0.0	0	0.0	36	100.0	1.000 [1.000; 1.000]	<0.001
Variants detected in the gene retrieved from draft WGS	618	100.0	0	0.0	0	0.0	36	100.0	1.000 [1.000; 1.000]	<0.001
Variants called from mapped short reads. at 80% frequency	618	100.0	0	0.0	0	0.0	36	100.0	1.000 [1.000; 1.000]	<0.001
Variants called from mapped short reads. at 0% frequency	615	100.0	0	0.0	3	7.7	36	92.3	0.958 [0.910; 1005]	<0.001
Ferric uptake regulator gene (*fur*)										
Variants detected in the consensus sequence	427	100.0	0	0.0	0	0.0	26	100.0	1.000 [1.000; 1.000]	<0.001
Variants detected in the gene retrieved from draft WGS	427	100.0	0	0.0	0	0.0	26	100.0	1.000 [1.000; 1.000]	<0.001
Variants called from mapped short reads. at 80% frequency	427	99.3	3	0.7	0	0.0	32	100.0	0.935 [0.862; 1.008]	<0.001
Variants called from mapped short reads. at 0% frequency	426	99.3	3	0.7	1	3.0	32	97.0	0.915 [0.833; 0.998]	<0.001

(*) The reference sequences used are wild-type genes obtained from the complete genome of reference strain 26695. The performance in detecting variants is judged based on gold standard sequences retrieved from the complete genome of the isolate DRC64. Tested sequences are those obtained from DRC64 based on the genes from the draft genome, the consensus sequences that were built, or the high-througH. *pylori*ut short reads that were mapped to a reference. Abbreviations are as follows: n, the number of counted alleles; TN, truly negative variants referring to alleles of wild-type that were identified across all loci in both the test and the gold standard sequences; FN, falsely negative variants corresponding to the alleles of wild-type detected in the test sequence instead of mutant alleles visible in the gold standard sequence; TP, truly positive variants that are mutant alleles detected in both the test and the gold standard sequences; FP, falsely positive alleles that are mutant alleles generated in the test sequence while they do not exist in the gold standard sequence.

Appendix B. Mutations detected in the putative AMR-encoding genes of *H. pylori* clinical isolates from the DRC

Mutations were reported following standard recommendations in molecular diagnostics from the Human Genome Variation Society [1, 2]. For a more thorough description of sequence variants, see https://varnomen.hgvs.org/. Briefly, a substitution with missense change (e.g., S402G) is described by the residue in the wild-type strain, the sequence-position, and the residue in the mutant strain. In-frame insertions (e.g., T337_S338insN) are described using "ins" after an indication of the residue flanking the insertion site, separated by a "_" (underscore) and followed by a description of the residue inserted. In-frame deletions (e.g., R131_K166del) are described using "del" after an indication of the first and last amino acid residues deleted separated by a "_" (underscore). A nonsense mutation or premature stop codon (e.g., W209Ter) is described like an amino acid substitution with "Ter" indicating an immediate translation stop codon. A frameshift mutation (e.g., Q65TfsTer10) is described using "fs" after the first amino acid affected by the mutational change, followed by the position of the translation termination codon (stop codon) in the new frame.

Nucleotide residues in DNA sequences are represented by heterocyclic bases as follows: A, Adenine; C, Cytosine; G, Guanine; and T, Thymine. Single-letter abbreviations for the amino acid residues in protein sequences are as follows: A, Alanine; C, Cysteine; D, Aspartic acid; E, Glutamic acid; F, Phenylalanine; G, Glycine; H, Histidine; I, Isoleucine; K, Lysine; L, Leucine; M, Methionine; N, Asparagine; P, Proline; Q, Glutamine; R, Arginine; S, Serine; T, Threonine; V, Valine; W, Tryptophan; and Y, Tyrosine.

Table S2. Potential genotypes of AMX-R encoded in *H. pylori* clinical isolates from DRC

**Genotype****	**Phenotypic AMX-S***				**Phenotypic AMX-R***				**p-value**
	**n_0**	**%**	**n_1**	**%**	**n_0**	**%**	**n_1**	**%**	
***pbp1A* gene**									
Mutations of the PBP-motif STGK$_{338_341}$	54	80.6	13	19.4	34	97.1	1	2.9	0.031
T337_S338insN	57	85.1	10	14.9	34	97.1	1	2.9	0.092
S338R	64	95.5	3	4.5	35	100.0	0	0.0	0.549
Mutations of the PBP-motif SAIK$_{368_371}$	65	97.0	2	3.0	29	82.9	6	17.1	0.019
F366L§	67	100.0	0	0.0	34	97.1	1	2.9	0.343
V374L	65	97.0	2	3.0	30	85.7	5	14.3	0.045
Mutations of the PBP-motif SKN$_{402_404}$	63	94.0	4	6.0	19	54.3	16	45.7	<0.001
Y401_S402insY	66	98.5	1	1.5	35	100.0	0	0.0	1.000
S402G#	65	97.0	2	3.0	24	68.6	11	31.4	<0.001
N404S	66	98.5	1	1.5	35	100.0	0	0.0	1.0000
S405N§	67	100.0	0	0.0	30	85.7	5	14.3	0.004
Mutations at codon S414 (S414R#)	67	100.0	0	0.0	33	94.3	2	5.7	0.116
Mutations at codon S455 (S455N)	65	97.0	2	3.0	35	100.0	0	0.0	0.545
Mutations at codon V469 (M/A)	62	92.5	5	7.5	30	85.7	5	14.3	0.305
V469M	62	92.5	5	7.5	32	91.4	3	8.6	1.000
V469A	67	100.0	0	0.0	33	94.3	2	5.7	0.116
Mutations at codon A474 (A474T§)	66	98.5	1	1.5	30	85.7	5	14.3	0.017
N504D	65	97.0	2	3.0	35	100.0	0	0.0	0.545

D535N	59	88.1	8	11.9	35	100.0	0	0.0	0.048
Mutations at codon S543 (H/R)	65	97.0	2	3.0	35	100.0	0	0.0	0.545
S543H	66	98.5	1	1.5	35	100.0	0	0.0	1.000
S543R	66	98.5	1	1.5	35	100.0	0	0.0	1.000
Mutations of the PBP-motif KTG$_{555_557}$	67	100.0	0	0.0	32	91.4	3	8.6	0.038
T556S[#]	67	100.0	0	0.0	32	91.4	3	8.6	0.038
Mutations at codon T558 (T558S[§])	67	100.0	0	0.0	30	85.7	5	14.3	0.004
Mutations of the PBP-motif SNN$_{559_561}$	55	82.1	12	17.9	17	48.6	18	51.4	0.001
Mutations at codon N562 (D/H/Y)	61	91.0	6	9.0	26	74.3	9	25.7	0.037
N562D	61	91.0	6	9.0	35	100.0	0	0.0	0.092
N562H[§]	67	100.0	0	0.0	34	97.1	1	2.9	0.343
N562Y[#]	67	100.0	0	0.0	27	77.1	8	22.9	<0.001
Mutations at codon T593 (A/G/K/P/S)	45	67.2	22	32.8	10	28.6	25	71.4	<0.001
T593A[#]	48	71.6	19	28.4	13	37.1	22	62.9	0.001
T593G	65	97.0	2	3.0	35	100.0	0	0.0	0.544
T593K	67	100.0	0	0.0	34	97.1	1	2.9	0.343
T593P	66	98.5	1	1.5	35	100.0	0	0.0	1.000
T593S	67	100.0	0	0.0	33	94.3	2	5.7	0.116
Mutations at codon G595 (del/A/S)	62	92.5	5	7.5	24	68.6	11	31.4	0.003
G595del	67	100.0	0	0.0	34	97.1	1	2.9	0.343
G595A	66	98.5	1	1.5	35	100.0	0	0.0	1.000
G595S	63	94.0	4	6.0	25	71.4	10	28.6	0.004
Mutations at codon A599	60	89.6	7	10.4	33	94.3	2	5.7	0.715
A599P	62	92.5	5	7.5	33	94.3	2	5.7	1.000
A599V	65	97.0	2	3.0	35	100.0	0	0.0	0.545
***pbp2* gene**									
Mutations of the PBP-motif SVVK$_{311_353}$	67	100	0	0	33	94.3	2	5.71	0.116
V312M	67	100.0	0	0.0	34	97.1	1	2.9	0.343
V313A	67	100.0	0	0.0	34	97.1	1	2.9	0.343
Mutations of the PBP-motif KTG$_{351_353}$	67	100.0	0	0.0	34	97.1	1	2.9	0.343
G353R	67	100.0	0	0.0	34	97.1	1	2.9	0.343
***pbp3* gene**									
Mutations of the PBP-motif SFN$_{232_234}$	67	100	0	0	34	97.1	1	2.86	0.343
F233L	67	100.0	0	0.0	34	97.1	1	2.9	0.343
***pbp4* gene**									
Mutations of the PBP-motif SYYK$_{265_268}$									
Y266H	66	98.5	1	1.5	35	100.0	0	0.0	1.000
Y267H	66	98.5	1	1.5	35	100.0	0	0.0	1.000

(*) n_0: No. of isolates without the genotype; n_1: No. of isolates encoding the genotype;

(**) Genotypes shown here were detected while screening all PBP-motifs (i.e., SXXK, SXN, and KTG motifs) and C-terminus codons of *pbp1A*, *pbp2*, *pbp3,* and *pbp4* genes. Mutations indicated are those detected in this study and are

categorized in three groups: AMX-R mutations previously proven experimentally by natural transformation (#); putative AMX-R mutations newly discovered in our strains (§); and putative AMX-R mutations that had been suspected previously, as summarized elsewhere [3, 4]. No additional putative AMX-R encoding genotype could be detected in *pbp* genes or even in *hofH* (i.e., G22W), *hefC* (i.e., D131E. L378F), or *hopC* (i.e., R302H) genes [3, 4].

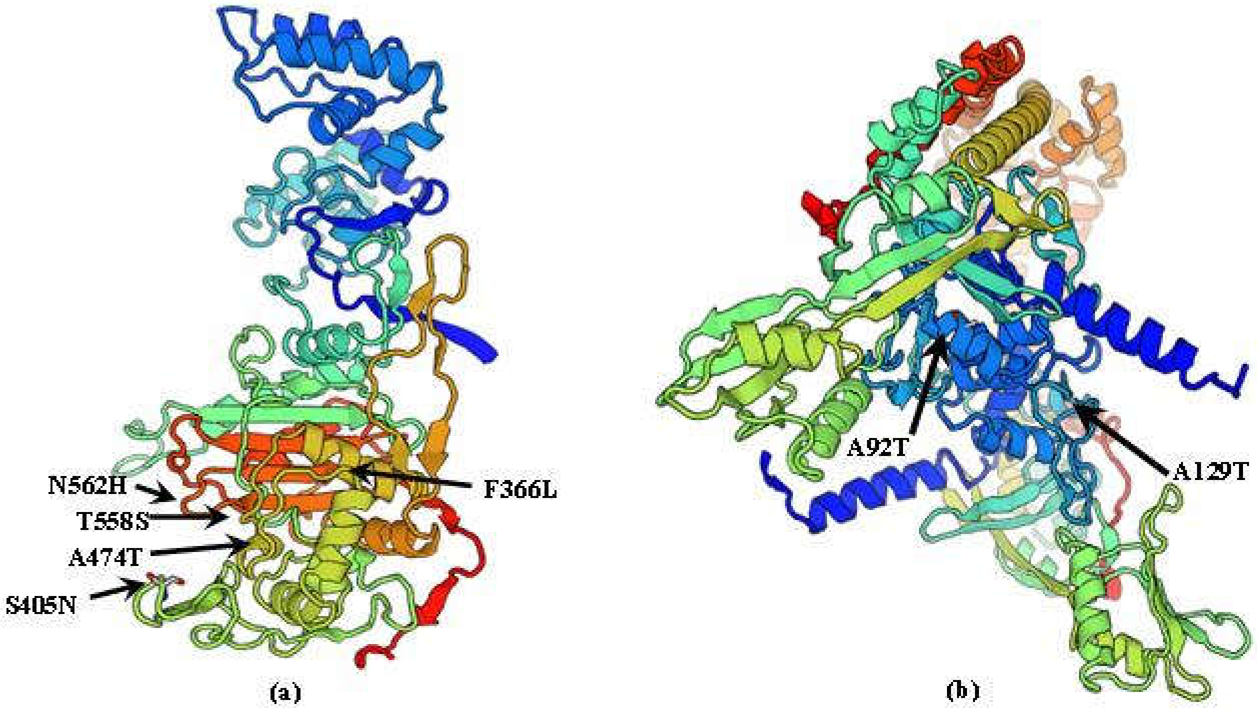

Figure S3. Putative AMX-R and LEV-R mutations newly discovered in this study.
This figure shows the structure of penicillin-binding protein 1A encoded by the *pbp1A* gene (panel (b)) and DNA gyrase subunits A encoded by the *gyrA* gene of 26695 (panel (a)) with corresponding putative AMR-related mutations newly discovered in this study. Mutational changes noted in the *pbp1A* gene resulted in amino acid substitutions altering penicillin-binding-motifs: F366L (altering the $SAIK_{368-371}$ motif in one AMR-R), S405N (adjoining the $SKN_{402-404}$ motif in five AMX-R strains), A474T (found in one AMX-S vs five AMX-R strains), T558S (between $KTG_{555-557}$ and $SNN_{559-561}$ motifs of five AMX-R strains), and N562H (adjoining $SNN_{559-561}$ in one AMX-R). New mutations found in *gyrA* resulted in A92T and A129T substitutions that fell in and outside the quinolone resistance-determining region (QRDR), respectively.

Table S3. Potential genotypes encoding CLA-R in *H. pylori* clinical isolates from DRC

Genotype**	Phenotypic CLA-S*				Phenotypic CLA-R*				p-value
	n_0	%	n_1	%	n_0	%	n_1	%	
***23S rRNA* gene**									
Mutations of the domain V	78	100.0	0	0.0	3	12.5	21	87.5	<0.001
A2142G	78	100.0	0	0.0	20	83.3	4	16.7	0.003
A2143G	78	100.0	0	0.0	7	29.2	17	70.8	<0.001
Mutations located outside the domain V	77	98.7	1	1.3	23	95.8	1	4.2	0.417
C2289T	77	98.7	1	1.3	23	95.8	1	4.2	0.417
***infB* gene**									
G160A	78	98.7	1	1.3	23	100.0	0	0.0	1.000

(*) n_0: No. of isolates without the genotype; n_1: No. of isolates encoding the genotype;
(**) Mutations are shown as nucleotide substitutions in the gene sequences. Genotypes indicated here were identified while screening the full-length *23S rRNA* gene to detect specific mutations that had been related to CLA-R (i.e., T1942C, G1939A, C2147G, G2172T, T2182C, A2116G, A2142G, A2143G, A2144G/T, A2115G, G2111A, A2142C, T2717C, T2289C, G2224A, and C2245T) [3, 4]. Only two nucleotide substitutions could thus be detected (i.e., A2142G, A2143G) in the domain V of *23 SrRNA* gene while no relevant mutation located outside this domain could be noted. No additional putative CLA-R-encoding genotype could be detected in the *rpl22* gene (i.e., T265_T266insTTCCATGTA and 226_228delGTG) in contrast to the *infB* gene, which showed a G160A mutation [5].

Table S4. Potential genotype encoding LEVO-R in *H. pylori* clinical isolates from DRC

Genotype**	Phenotypic LEVO-S*				Phenotypic LEVO-R*				p-value
	n_0	%	n_1	%	n_0	%	n_1	%	
***gyrA* gene**									
Mutations in and outside QRDR	18	51.4	17	48.6	4	6	63	94	<0.0001
Mutations of the QRDR (A71 to Q110)	32	91.4	3	8.6	7	10.4	60	89.6	<0.0001
Mutations at codon N87	34	97.1	1	2.9	31	46.3	36	53.7	<0.0001
N87I	35	100	0	0	46	68.7	21	31.3	0.0001
N87K	35	100	0	0	58	86.6	9	13.4	0.0259
N87T	34	97.1	1	2.9	61	91	6	8.96	0.4172
Mutations at codon D91	34	97.1	1	2.9	43	64.2	24	35.8	0.0002
D91G	35	100	0	0	59	88.1	8	11.9	0.0483
D91N	34	97.1	1	2.9	52	77.6	15	22.4	0.0095
D91Y	35	100	0	0	66	98.5	1	1.49	1.0000
Mutations at codon A92 (A92T§)	34	97.1	1	2.9	60	89.6	7	10.4	0.2580
Mutations at codon R103 (R103H)	35	100	0	0	66	98.5	1	1.49	1.0000
Mutations located outside the QRDR	21	60	14	40	46	68.7	21	31.3	0.3899
Mutations at codon A129 (A129T)	35	100	0	0	66	98.5	1	1.49	1.0000
Mutations at codon R130 (R130K)	23	65.7	12	34.3	59	88.1	8	11.9	0.0095
Mutations at codon A199 (A199V)	33	94.3	2	5.71	54	80.6	13	19.4	0.0804

***gyrB* gene**									
Mutations in and outside QRDR	33	94.3	2	5.7	58	86.6	9	13.4	0.3229
Mutations of the QRDR (E415 to S454)	35	100.0	0	0.0	65	97	2	3.0	0.5447
D435N	35	100.0	0	0.0	66	98.5	1	1.5	1.0000
V437L	35	100.0	0	0.0	66	98.5	1	1.5	1.0000
Mutations located outside the QRDR	33	94.3	2	5.7	60	89.6	7	10.4	0.7148
R484K	34	97.1	1	2.9	67	100	0	0.0	0.3431
R579C[§]	34	97.1	1	2.9	60	89.6	7	10.4	0.2580

(*) n_0: No. of isolates without the genotype; n_1: No. of isolates encoding the genotype;
(**) Genotypes of LEVO AST were detected while screening full-length *gyrA* and *gyrB* genes. QRDR stands for quinolone resistance-determining region. Mutations shown are those detected in this study among mutations previously related to LEVO-R in DNA gyrase A (i.e., H57Y, S63P, V65I, V77A, S83A, D86N, N87A/K/I/Y, A88N/P/V, D91G/N/A/H/Y, D99V, A129T, R130K, D155N, D161N, V172I, P188S, D192N, and V199A/I) and in DNA gyrase B (i.e., D481E, R484K, F438S, S429T, and E463K) [3, 4] but also include putative LEVO-R mutations newly discovered in our strains ([§]).

Table S5 (A). Potential genotype encoding MTZ-R in *H. pylori* clinical isolates from DRC

Genotype**	Phenotypic MTZ-S*				Phenotypic MTZ-R*				p-value
	**n_0**	**%**	**n_1**	**%**	**n_0**	**%**	**n_1**	**%**	
***rdxA* gene**									
Wild type sequence	4	40.0	6	60.0	79	85.9	13	14.1	0.003
Mutations at a known functional loci	9	90.0	1	10.0	25	27.2	67	72.8	0.000
Null mutations	10	100.0	0	0.0	44	47.8	48	52.2	0.001
Frameshift mutations	10	100.0	0	0.0	67	72.8	25	27.2	0.114
Q65TfsTer10	10	100.0	0	0.0	85	92.4	7	7.6	1.000
S79AfsTer5	10	100.0	0	0.0	89	96.7	3	3.3	1.000
A193GfsTer13	10	100.0	0	0.0	90	97.8	2	2.2	1.000
G162WfsTer4	10	100.0	0	0.0	90	97.8	2	2.2	1.000
D59IfsTer4	10	100.0	0	0.0	91	98.9	1	1.1	1.000
E194RfsTer12	10	100.0	0	0.0	91	98.9	1	1.1	1.000
F117LfsTer7	10	100.0	0	0.0	91	98.9	1	1.1	1.000
H69PfsTer6	10	100.0	0	0.0	91	98.9	1	1.1	1.000
K190EfsTer15	10	100.0	0	0.0	91	98.9	1	1.1	1.000
Q50RfsTer7	10	100.0	0	0.0	91	98.9	1	1.1	1.000
R41KfsTer20	10	100.0	0	0.0	91	98.9	1	1.1	1.000
S116LfsTer43	10	100.0	0	0.0	91	98.9	1	1.1	1.000
W52HfsTer5	10	100.0	0	0.0	91	98.9	1	1.1	1.000
Y26TfsTer9	10	100.0	0	0.0	91	98.9	1	1.1	1.000
Y71_F72insSfsTer5	10	100.0	0	0.0	91	98.9	1	1.1	1.000
Premature stop codons	10	100.0	0	0.0	80	87.0	12	13.0	0.602
Q50Ter	10	100.0	0	0.0	87	94.6	5	5.4	1.000

K181Ter	10	100.0	0	0.0	91	98.9	1	1.1	1.000
L153Ter	10	100.0	0	0.0	91	98.9	1	1.1	1.000
L33Ter	10	100.0	0	0.0	91	98.9	1	1.1	1.000
Q102Ter	10	100.0	0	0.0	91	98.9	1	1.1	1.000
R112Ter	10	100.0	0	0.0	91	98.9	1	1.1	1.000
W209Ter	10	100.0	0	0.0	91	98.9	1	1.1	1.000
W52Ter	10	100.0	0	0.0	91	98.9	1	1.1	1.000
Large sequence deletions	10	100.0	0	0.0	82	89.1	10	10.9	0.592
K2_M21del§	10	100.0	0	0.0	89	96.7	3	3.3	1.000
R131_K166del§	10	100.0	0	0.0	89	96.7	3	3.3	1.000
K168_V172del§	10	100.0	0	0.0	91	98.9	1	1.1	1.000
L137_I142del§	10	100.0	0	0.0	91	98.9	1	1.1	1.000
N178_L185del; G189_R200del§	10	100.0	0	0.0	91	98.9	1	1.1	1.000
S92_Q146del§	10	100.0	0	0.0	91	98.9	1	1.1	1.000
Large sequence insertions ending with a stop	10	100.0	0	0.0	91	98.9	1	1.1	1.000
K168_V169insSGRDFRTAYQTer§	10	100.0	0	0.0	91	98.9	1	1.1	1.000
Point-mutations at functional codons	9	90.0	1	10.0	64	69.6	28	30.4	0.274
R16C/H	10	100.0	0	0.0	86	93.5	6	6.5	1.000
H97T/Y	10	100.0	0	0.0	87	94.6	5	5.4	1.000
S43L	10	100.0	0	0.0	89	96.7	3	3.3	1.000
A118S	10	100.0	0	0.0	89	96.7	3	3.3	1.000
S108A	10	100.0	0	0.0	90	97.8	2	2.2	1.000
G145R/W	10	100.0	0	0.0	90	97.8	2	2.2	1.000
K203C/E	10	100.0	0	0.0	90	97.8	2	2.2	1.000
H17Y	10	100.0	0	0.0	91	98.9	1	1.1	1.000
S18P	10	100.0	0	0.0	91	98.9	1	1.1	1.000
C19Y	10	100.0	0	0.0	91	98.9	1	1.1	1.000
E27A	10	100.0	0	0.0	91	98.9	1	1.1	1.000
R41K	10	100.0	0	0.0	91	98.9	1	1.1	1.000
R90N	10	100.0	0	0.0	91	98.9	1	1.1	1.000
P106S	10	100.0	0	0.0	91	98.9	1	1.1	1.000
I142del	10	100.0	0	0.0	91	98.9	1	1.1	1.000
C148Y	10	100.0	0	0.0	91	98.9	1	1.1	1.000
G163D/V	9	90.0	1	10.0	91	98.9	1	1.1	0.187
R200K	10	100.0	0	0.0	91	98.9	1	1.1	1.000

(*) n_0: No. of isolates without the genotype; n_1: No. of isolates encoding the genotype

(**) Genotypes indicated here were detected while screening the full-length *rdxA* gene to detect specific mutations at functional codons well-defined elsewhere [6] (e.g., R16, H17, S18, C19, K20, R41, L42, S43, Y47, Q50, V55, M56, N73, I142, A143, G145, G149, C159, G162, G163, V192, K198, K200, K202, L209). In addition to changes altering these loci, mutations that had been suspected in the MTZ-R of the clinical isolates were also searched (e.g., A22S, E27Q/V, T31E, D59N, R90K, H97T/Y, P106S, S108A, A118S/T, R131K, and G189C) [3, 4]. Putative MTZ-R-encoding mutations newly described in this study are also reported (§).

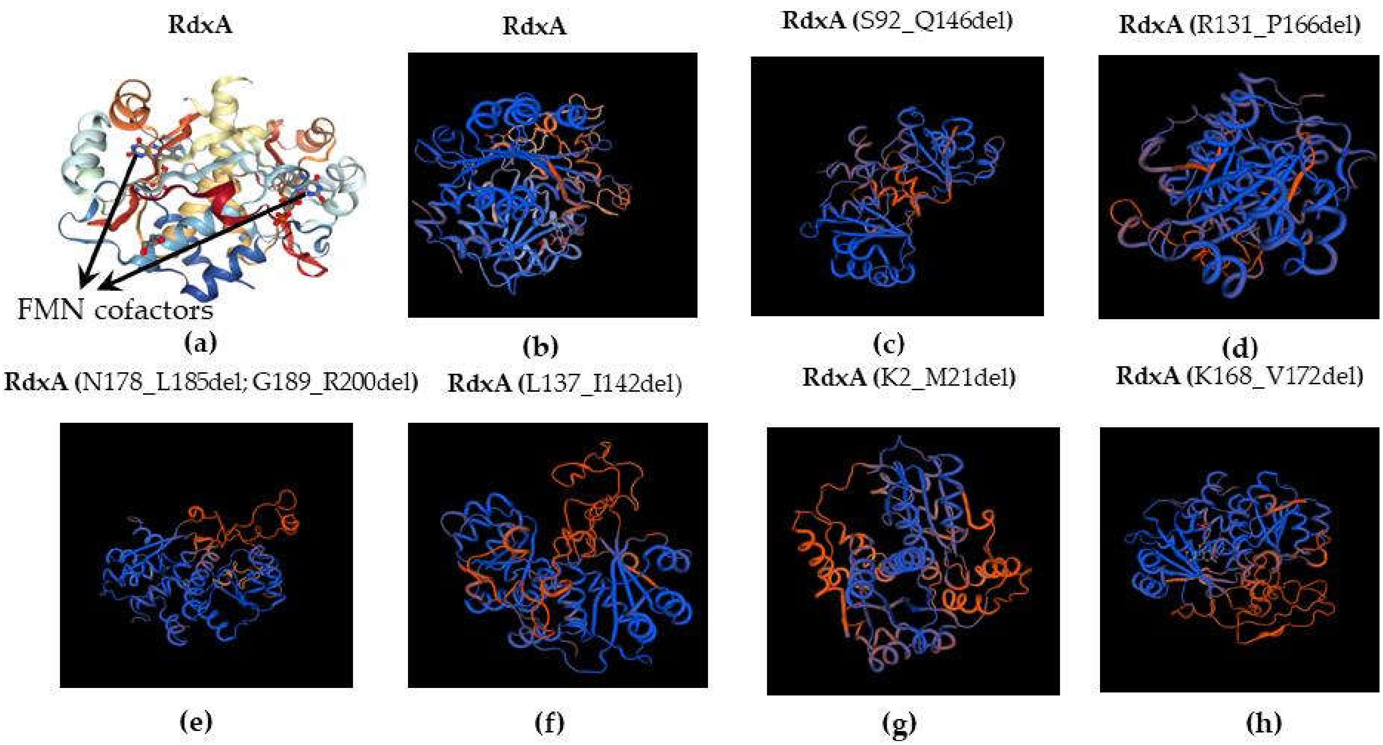

Figure S4. Protein structure of the oxygen-insensitive NAD(P)H nitroreductase encoded by the *rdxA* gene.

The crystal structure of RdxA has been fully described elsewhere [3]. Panel (a) shows the 3D structure of the RdxA protein of *H. pylori* that is publicly available at http://www.rcsb.org/3d-view/3QDL. Typically, each monomer of RdxA binds two flavin mononucleotide (FMN) molecules that are important cofactors for MTZ-reductive activation by the apoprotein [3]. Panel (b) shows the 3D structure inferred for an RdxA protein of an MTZ-susceptible *H. pylori* isolate from the DRC. Panels (c) to (h) represent the structure of RdxA proteins found with various sequence deletions in this study. It appears that RdxA molecules from (c) to (h) have lost their ability to bind FMN cofactors owing to a lack of binding sites. As indicated on panel (h), the affinity for FMN molecules is partially reduced as only one cofactor can still be bound to the protein. These deletions are thus expected to induce MTZ-R basically owing to a loss of affinity for FMN cofactor [3].

Table S6 (B). Potential genotype encoding MTZ-R in *H. pylori* clinical isolates from DRC

Genotype**	Phenotypic MTZ-S*				Phenotypic MTZ-R*				p-value
	n_0	%	n_1	%	n_0	%	n_1	%	
***frxA* gene**									
Wild type sequence	10	100.0	0	0.0	88	95.7	4	4.3	1.000
Mutations at a known functional loci	6	60.0	4	40.0	55	59.8	37	40.2	1.000
Null mutations	6	60.0	4	40.0	58	63.0	34	37.0	1.000
Frameshift mutations	10	76.9	3	23.1	82	80.4	20	19.6	0.690
A70HfsTer30	10	100.0	0	0.0	90	97.8	2	2.2	1.000
A70RfsTer5	10	100.0	0	0.0	91	98.9	1	1.1	1.000
E164GfsTer4	10	100.0	0	0.0	91	98.9	1	1.1	1.000
E200KfsTer16	9	90.0	1	10.0	92	100.0	0	0.0	1.000
E214GfsTer4	10	100.0	0	0.0	91	98.9	1	1.1	1.000
F72LfsTer3	10	100.0	0	0.0	91	98.9	1	1.1	1.000
G179KfsTer4	10	100.0	0	0.0	91	98.9	1	1.1	1.000
K18NfsTer16	9	90.0	1	10.0	90	97.8	2	2.2	1.000
M191IfsTer8	10	100.0	0	0.0	91	98.9	1	1.1	1.000
P48HfsTer7	10	100.0	0	0.0	91	98.9	1	1.1	1.000
R23LfsTer15	10	100.0	0	0.0	91	98.9	1	1.1	1.000
R24LfsTer16	9	90.0	1	10.0	88	95.7	4	4.3	1.000
S112GfsTer21	10	100.0	0	0.0	91	98.9	1	1.1	1.000
T205NfsTer10	10	100.0	0	0.0	90	97.8	2	2.2	1.000
T205PfsTer11	10	100.0	0	0.0	91	98.9	1	1.1	1.000
Premature stop codons	9	90.0	1	10.0	78	84.8	14	15.2	1.000
D92Ter	10	100.0	0	0.0	90	97.8	2	2.2	1.000
E2014Ter	10	100.0	0	0.0	91	98.9	1	1.1	1.000
E57Ter	10	100.0	0	0.0	91	98.9	1	1.1	1.000
E61Ter	9	90.0	1	10.0	92	100.0	0	0.0	1.000
G73Ter	10	100.0	0	0.0	89	96.7	3	3.3	1.000
Q199Ter	10	100.0	0	0.0	91	98.9	1	1.1	1.000
R86Ter	10	100.0	0	0.0	91	98.9	1	1.1	1.000
W137Ter	10	100.0	0	0.0	90	97.8	2	2.2	1.000
W207Ter	10	100.0	0	0.0	90	97.8	2	2.2	1.000
W30Ter	10	100.0	0	0.0	91	98.9	1	1.1	1.000
Functional point mutations	10	100.0	0	0.0	89	96.7	3	3.3	1.000
G165V	10	100.0	0	0.0	91	98.9	1	1.1	1.000
R206H	10	100.0	0	0.0	90	97.8	2	2.2	1.000
***fur* gene**									
Wild types *fur* sequence	0	0.0	10	100.0	3	3.3	89	96.7	1.000
Functional point mutation	10	100.0	0	0.0	91	94.8	1	1.1	1.000

P114S	10	100.0	0	0.0	91	98.9	1	1.1	1.000
sodB promoter region	10	100.0	0	0.0	91	98.9	1	1.1	1.000
A-5C	10	100.0	0	0.0	91	98.9	1	1.1	1.000

(*) n_0: No. of isolates without the genotype; n_1: No. of isolates encoding the genotype

(**) Genotypes indicated here were detected while screening the full-length *rdxA* and *fur* genes as well as the promoter region of the sodB gene to detect specific mutations at functional codons well-defined elsewhere for FrxA (e.g., K17, R13, A15, K20, Q164, G165, R206) [6], for Fur (e.g., R3, M42, Y65, C78, E90, H99, E110, P114, and HHDHXXCXXC$_{96_105}$-motif) [7, 8], and for the sodB promoter region (i.e., A-5C) [8]. No mutation previously related to phenotypic MTZ-R could be observed in the *recA* gene encoding a protein involved in DNA recombination and repair (i.e., Y103H and S121D), in *mdaB* encoding the modulator of drug activity (i.e., R99I and G98D), in *ribF* gene of riboflavin (i.e., T222M and A227T), in the *omp11* gene of outer membrane protein 11 (i.e., A1290D), and in the *rpsU* gene of 30S ribosomal protein S21 (i.e., D13T**)**.

References

1. Den Dunnen J, Antonarakis S: Nomenclature for the description of human sequence variations. Human genetics 2001, 109(1):121-124.
2. Ogino S, Gulley ML, Den Dunnen JT, Wilson RB, Training AfMP, Committee E: Standard mutation nomenclature in molecular diagnostics: practical and educational challenges. The Journal of molecular diagnostics 2007, 9(1):1-6.
3. Gong Y, Yuan Y: Resistance mechanisms of *Helicobacter pylori* and its dual target precise therapy. Critical reviews in microbiology 2018, 44(3):371-392.
4. Hu Y, Zhang M, Lu B, Dai J: *Helicobacter pylori* and antibiotic resistance, a continuing and intractable problem. Helicobacter 2016, 21(5):349-363.
5. Binh TT, Shiota S, Suzuki R, Matsuda M, Trang TTH, Kwon DH, Iwatani S, Yamaoka Y: Discovery of novel mutations for clarithromycin resistance in *Helicobacter pylori* by using next-generation sequencing. Journal of Antimicrobial Chemotherapy 2014, 69(7):1796-1803.
6. Martínez-Júlvez M, Rojas AL, Olekhnovich I, Angarica VE, Hoffman PS, Sancho J: Structure of R dx A–an oxygen-insensitive nitroreductase essential for metronidazole activation in H elicobacter pylori. The FEBS journal 2012, 279(23):4306-4317.
7. Choi SS, Chivers PT, Berg DE: Point mutations in *Helicobacter pylori*'s fur regulatory gene that alter resistance to metronidazole, a prodrug activated by chemical reduction. PLoS One 2011, 6(3).
8. Tsugawa H, Suzuki H, Satoh K, Hirata K, Matsuzaki J, Saito Y, Suematsu M, Hibi T: Two amino acids mutation of ferric uptake regulator determines *Helicobacter pylori* resistance to metronidazole. Antioxidants & redox signaling 2011, 14(1):15-23.
